# Differential and Prognostic Significance of HOXB7 in Gliomas

**DOI:** 10.3389/fcell.2021.697086

**Published:** 2021-08-11

**Authors:** Xingang Zhou, Tingyu Liang, Jinhai Deng, Kenrick Ng, Man Li, Chunxin Lv, Jiamin Chen, Kun Yang, Zhiyuan Ma, Wenping Ma, Peng Wang

**Affiliations:** ^1^Department of Pathology, Beijing Ditan Hospital, Capital Medical University, Beijing, China; ^2^Department of Neurosurgery, Beijing Ditan Hospital, Capital Medical University, Beijing, China; ^3^Key Laboratory of Medical Immunology, School of Basic Medical Sciences, Peking University Center for Human Disease Genomics, Peking University Health Science Center, Beijing, China; ^4^Richard Dimbleby Department of Cancer Research, Comprehensive Cancer Center, Kings College London, London, United Kingdom; ^5^Department of Medical Oncology, University College London Cancer Institute, London, United Kingdom; ^6^Geriatric Department, Minhang Hospital, Fudan University, Shanghai, China; ^7^Department of Neurosurgery, Beijing Tiantan Hospital, Capital Medical University, Beijing, China

**Keywords:** oligodendroglioma, astrocytoma, HOXB7, immunohistochemistry, prognosis

## Abstract

Diffuse glioma is the most common primary tumor of the central nervous system. The prognosis of the individual tumor is heavily dependent on its grade and subtype. Homeobox B7 (HOXB7), a member of the homeobox family, is abnormally overexpressed in a variety of tumors. However, its function in glioma is unclear. In this study, HOXB7 mRNA and protein expression levels were analyzed in 401 gliomas from the CGGA RNA-seq database (325 cases) and our hospital (76 cases). HOXB7 expression, at both mRNA and protein levels, were upregulated in glioblastoma (GBM) and isocitrate dehydrogenase 1 (*IDH1*) wild-type glioma tissues. Kaplan–Meier with log-rank test showed that patients with high HOXB7 expression had a poor prognosis (*p* < 0.0001). Moreover, HOXB7 protein was deleted in 90.9% (20/22) of oligodendrogliomas and 13.0% (3/23) of astrocytomas. The sensitivity and specificity of HOXB7 protein deletion in oligodendroglioma were 90.9% (20/22) and 87.0% (20/23), respectively. To verify the reliability of using HOXB7 in differentiating oligodendroglioma, we used 1p/19q fluorescence *in situ* hybridization (FISH) testing as a positive control. The Cohen’s kappa coefficient of HOXB7 immunohistochemistry staining and 1p/19q FISH testing was 0.778 (95% CI: 0.594–0.962, *p* < 0.001). In conclusion, HOXB7 is an independent predictor of poor prognosis in all grade gliomas. Additionally, HOXB7 is also a highly sensitive and specific indicator to differentiate oligodendroglioma from astrocytoma.

## Introduction

Diffuse gliomas encompass a broad range of tumors affecting the central nervous system. These include astrocytomas, oligodendrogliomas, and oligoastrocytomas, which range from Grade II to Grade IV according to the World Health Organization (WHO) classification system. They account for approximately 80% of the primary malignant tumors in the central nervous system. The prognosis and management of the individual tumors is variable, and highly dependent on the grade and subtype. Grade II and III astrocytoma and oligodendroglioma are the two main histological types of lower grade gliomas (LGG), accounting for 43.2% of all gliomas diagnosed in adults (Stupp et al., 2005; Louis et al., 2016; Hu et al., 2018). The 2016 classification of CNS tumors introduced “integrated diagnosis” combining histological and molecular genetic information, with the purpose of improving the accuracy of prognostic analysis and to guide clinical diagnosis and treatment more effectively (Louis et al., 2016). Isocitrate dehydrogenase 1 (*IDH1*) mutation, which occurs early in gliomagenesis particularly in WHO grade II and III gliomas, is an acknowledged molecular alteration (Binder et al., 2019). The demonstration of both IDH gene family mutation and combined whole-arm losses of 1p and 19q (1p/19q codeletion) is characteristic of oligodendrogliomas (Louis et al., 2016). However, many institutions are unable to conduct such comprehensive molecular testing. Within this clinical setting, there is an urgent need to identify specific markers for alternative diagnosis strategies for human glioma.

Homeobox B7 (HOXB7), a member of the homeobox family abnormally overexpressed in a variety of solid tumors, plays an important role in proliferation, migration and invasion of tumor cells (Carè et al., 2001; Kanai et al., 2010; Chile et al., 2013; Heideman et al., 2015; He et al., 2017; Huan et al., 2017; Dai et al., 2019; Zhou et al., 2020). Its expression is closely related to clinicopathological features, but the signaling pathways causing this phenotype have yet to be elucidated. Molecular genetic studies have shown that the homeobox family may be associated with the occurrence and development of glioma (Samuel and Naora, 2005). Several reports have suggested that high expression of homeotic genes, including HOXA9 (Costa et al., 2010), HOXA13 (Duan et al., 2015), mesenchyme HOX2 (MEOX2) (Tachon et al., 2019), and HOXD4 (Zhao et al., 2017) is an indicator of poor prognosis in glioblastoma (GBM) patients. Our latest research also showed that HOXB8, another member of the homeobox B family, is a crucial contributor to the aggressiveness of GBM (Ma et al., 2020), but the expression of HOXB7 in human glioma remains uncharacterized.

In this study, we included 401 glioma patients from the CGGA RNA-seq database (325 cases) and our patient cohort (76 cases) from Ditan Hospital. The mRNA and protein expression levels of HOXB7 in different clinical and molecular glioma subtypes were analyzed. We also examined the prognostic value of HOXB7 in glioma patients with different WHO grades. In addition, we evaluated the sensitivity and specificity of HOXB7 as a marker to differentiate oligodendrogliomas from astrocytomas.

## Materials and Methods

### Patients and Samples

**FIGURE 1:**
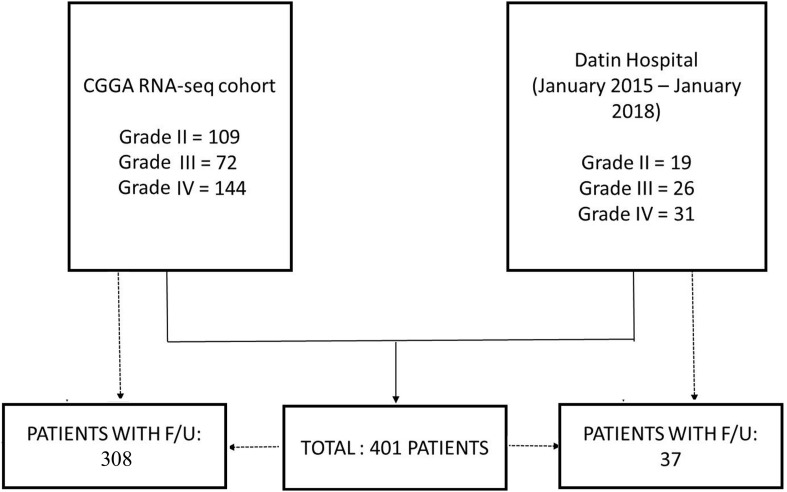
CONSORT-like diagram of patients enrolled in the study.

A total of 325 patients with WHO grade II–IV glioma (325 cases from the CGGA RNA-seq cohort) were included in this study (Figure 1). The CGGA RNA-seq cohort^1^ comprised of 109 grade II, 72 grade III, and 144 grade IV patients. 308 patients had appropriate follow-up, including 101 grade II (45 cases of astrocytoma *IDH1* mutant type, 13 cases of astrocytoma *IDH1* wild type and 43 cases of oligodendrogliomas 1p/19q codeletion), 71 grade III (27 cases of astrocytoma *IDH1* mutant type, 28 cases of astrocytoma *IDH1* wild type and 16 cases of oligodendrogliomas 1p/19q codeletion), and 136 grade IV (40 cases of *IDH1* mutant type and 96 cases of *IDH1* wild type) patients. Data from an additional 76 patients who underwent neurosurgery in our hospital from January 2015 to January 2018 were analyzed retrospectively. These 76 clinical specimens comprised of 19 grade II (8 cases of astrocytoma *IDH1* mutant type, 2 cases of astrocytoma *IDH1* wild type and 9 cases of oligodendrogliomas 1p/19q codeletion), 26 grade III (5 cases of astrocytoma *IDH1* mutant type, 8 cases of astrocytoma *IDH1* wild type and 13 cases of oligodendrogliomas 1p/19q codeletion), and 31 grade IV gliomas (4 cases of *IDH1* mutant type and 27 cases of *IDH1* wild type). The histological and molecular diagnoses were determined according to the WHO 2016 criteria (Louis et al., 2016). Among them, 37 patients had appropriate follow-up. Overall survival (OS) ranged from 2 to 62 months, defined as the period from surgery to death (Yang et al., 2016).

### Bioinformatic Analysis

Prior to analysis, the raw data was normalized by log transformation. OS was defined as the time between diagnosis and death or missing information in the CGGA database. These data were calculated and classified into high and low expression groups by the median value of HOXB7. We assessed the prognostic value of HOXB7 in the different subgroups by using Kaplan–Meier survival curves with log-rank test. Univariate and multivariate Cox regression analyses were used to seek out the independent prognostic factors.

### Immunohistochemistry

The expression of HOXB7 protein was assessed by immunohistochemistry. The tumor tissues excised during the operation were immediately placed in 10% formalin for fixation, followed by dehydration, paraffin embedding, and sectioning. Anti-HOXB7 antibody (SAB1412447, Sigma-Aldrich, United States) was used at a dilution of 1:1,500. All tissue sections were incubated with primary antibody at 4°C overnight. An EnVision Chem Detection Kit (DaKo Cytomation, CA) secondary antibody was used at room temperature for 30 min. The results were interpreted by two neuropathologists independently. HOXB7 protein expression (semi-quantitative scoring, SQS) = expression intensity × expression area. Expression intensity was scored from 0 to 3 (at 10 × 20 magnification, 5 different fields of view were selected randomly and observed under the microscope; the average of the combined counts of 5 fields was calculated), representing negative, weakly staining (light yellow), moderately staining (pale brown with light background), and strongly staining (dark brown without background), respectively. Expression area was scored from 0 to 4 (at 10 × 10 magnification the total positive area was observed and evaluated), representing <5, 6–25, 26–50, 51–75, and >75%, respectively. The degree of positive staining: 1–3 was classified as weakly positive (+); 4–6 as moderately positive (++); and 7–12 as strongly positive (+++) (Feng et al., 2019).

### Fluorescence *in situ* Hybridization

Fluorescence *in situ* hybridization (FISH) was performed to detect the 1p/19q codeletion status in all LGG cases. The 1p/19q probe (F.01081-01; GSP 1p36/GSP 1q21, GSP 19q13/GSP 19p13) was purchased from GuangZhou LBP Medicine Science and Technology Co. Ltd. (China). Briefly, deparaffinized sections were pretreated by pressure cooking for 20 min and incubated in pepsin solution at 37°C for 20 min. After dehydration through an alcohol gradient and air drying, probes were added to tissue sections, denatured at 85°C for 5 min and hybridized overnight at 37°C. Finally, the sections were washed and counter stained with 4′, 6-diamidino-2-phenylindole (DAPI) then mounted with coverslips.

### *IDH* Gene Status

*IDH1* R132H analysis was confirmed by IHC and DNA sequencing. Paraffin sections of the glioma tumor specimens were stained with IDH1 R132H mutation-specific antibody (working solution, H09 clone, ZSGB-BIO, China). DNA sequencing was performed when the IHC results were negative. The IDH1 forward primer (5′-ACC AAA TGG CAC CAT ACG A-3′) and reverse primer (5′-GCA AAATCA CAT TAT TGC CAA C-3′) were designed to amplify exon 4 (codon R132) of the IDH1 gene (Nagashima et al., 2016). The PCR template comprised a denaturing step lasting 5 min at 95°C followed by 35 amplification cycles of 95°C for 45 s, 56°C for 45 s, 72°C for 45 s, and a final extension step at 72°C for 7 min.

### Statistical Analysis

R programming language, GraphPad Prism 5, and SPSS 21.0 were used for statistical analyses. Student’s *t*-test was used to explore the diversity of HOXB7 expression between different clinical and molecular groups. Correlation between HOXB7 expression level and WHO grade was analyzed using the Pearson’s correlation test. Kaplan–Meier analysis was performed to estimate the survival time of different subgroups, and a log-rank test assessed the prognostic differences. Univariate and multivariate Cox regression analyses were used to determine that HOXB7 is an independent factor to predict survival. *p* < 0.05 was considered as statistically significant.

## Results

### Homeobox B7 Expression Is Upregulated in GBM and *IDH1* Wild-Type Glioma

**FIGURE 2:**
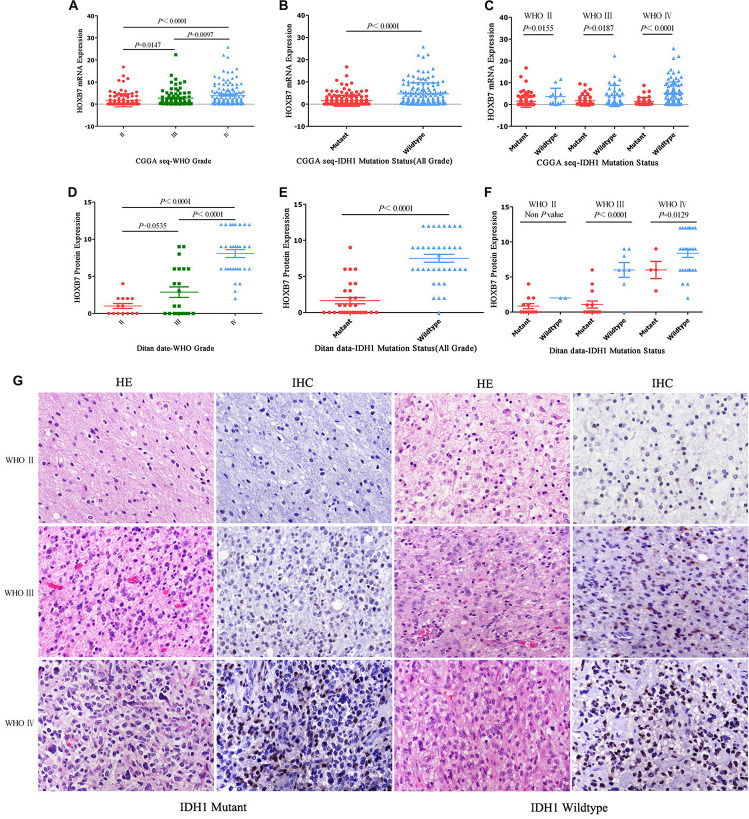
Homeobox B7 (HOXB7) expression pattern in CGGA RNA-seq cohort and Ditan Hospital cohort. HOXB7 is enriched in high-grade gliomas in CGGA RNA-seq database **(A)** and Ditan Hospital database **(D)**. HOXB7 is enriched in IDH1-wildtype gliomas in CGGA RNA-seq database **(B,C)** and Ditan Hospital database **(E,F)**. Immunohistochemical staining of HOXB7 protein expression in different WHO grade gliomas [**(G)**, HE, EnVision, serial sections, Original magnification ×400]. HOXB7 protein is located in the nucleus of tumor cells. With the increase of WHO grade, the number of positive tumor cells increased as well as the enhanced intensity of positive signal. In the same WHO grade glioma, the HOXB7 protein was expressed more highly in *IDH1* wild type than that in *IDH1* mutant type. The semi-quantitative scoring (expression intensity × expression area) of each specimen are as follows. WHO II and *IDH1* Mutant: (1 × 1 = 1), WHO II and *IDH1* Wild: (2 × 1 = 2), WHO III and *IDH1* Mutant: (2 × 2 = 4), WHO III and *IDH1* Wild: (2 × 3 = 6), WHO IV and *IDH1* Mutant: (3 × 2 = 6), WHO IV and *IDH1* Wild: (3 × 3 = 9).

First, we investigated the expression pattern of HOXB7 in gliomas by analyzing the mRNA expression level of *HOXB7* based on the RNA-seq data of 325 samples from the CGGA database. The results showed that the HOXB7 mRNA level was positively associated with WHO grade (*R* = 0.316, *p* < 0.001, Figure 2A) by Pearson’s correlation test. In both WHO all-grade gliomas and within the same WHO grade gliomas, the level of *HOXB7* mRNA was more highly expressed in *IDH1* wild type gliomas than in *IDH1* mutant types (*p* < 0.0001, Figures 2B,C). To validate the results above at the protein level, the expression of HOXB7 protein in paraffin embedded tissues was assessed by IHC. The clinicopathologic characteristics of the 76 cases from our hospital database are shown in Table 1. As anticipated, the expression trend of HOXB7 protein was consistent with that of RNA level in different WHO grades or molecular types of gliomas (*R* = 0.728, *p* < 0.001, Figures 2D–F).

**TABLE 1:** Clinical characteristics and homeobox B7 (HOXB7) protein expression.

Parameters		Total	HOXB7 expression		IHC SQS	*P*-value
		*n* = 76	Positive (*n*)	Negative (*n*)	(0~12)	
Age (mean/median)		45/46	46/48	43/45	–	0.614
Gender						
Male		44	31	13	5 ± 0.68	0.783
Female		32	22	10	4.71 ± 076	
Oligodendrocytoma						
WHO II	IDH mut	9	0	9	0	–
	IDH wt	0	0	0	0	–
WHO III	IDH mut	13	2	11	0.18 ± 0.12	0.341*
	IDH wt	0	0	0	0	–
Astrocytoma						
WHO II	IDH mut	8	6	2	1.43 ± 0.53	0.048*
	IDH wt	2	2	0	2	–
WHO III	IDH mut	5	5	0	4.33 ± 0.88	0.001*
	IDH wt	8	7	1	6 ± 1.05	0.001*
GBM (WHO IV)	IDH mut	4	4	0	6 ± 1.22	0.001*
	IDH wt	27	27	0	8.37 ± 0.58	0.001*

*n, number of cases; wt, wild type; mut, mutant; IHC SQS, Immunohistochemistry semi-quantitative scoring; GBM, Glioblastoma, ^∗^, student’s *t*-test, each group was compared with Oligodendrocytoma (WHO II IDH mut) group.*

Figure 2G is an intuitive display of the expression of HOXB7 protein. The positive signal was located in the nucleus. With increasing WHO grade, the number of positive tumor cells increased as well as the intensity of positive signal. In the same WHO grade glioma, the HOXB7 protein was expressed more highly in *IDH1* wild type than that in *IDH1* mutant type.

### High Homeobox B7 Expression Confers Reduced Survival Time in All Grade and Lower Grade Glioma

**FIGURE 3:**
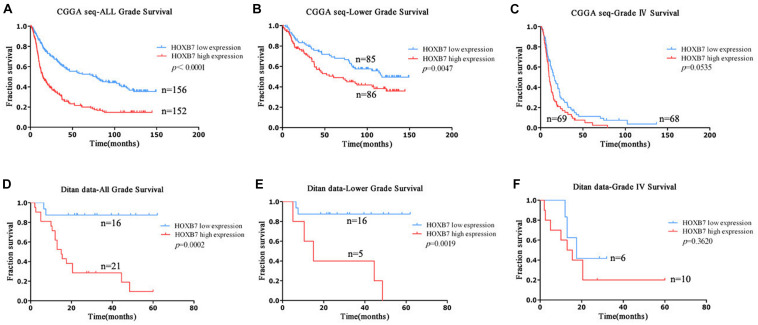
Prognostic significance of HOXB7 in all-grade, lower-grade and GBM groups. **(A–C)** CGGA RNA-seq database; **(D–F)** Ditan database.

To further explore the above findings, clinical outcomes of patients with gliomas were collected. HOXB7-specific Kaplan–Meier survival analysis of the clinical cohort revealed that high HOXB7 expression was associated with poor OS (Figure 3). The patients, who were from the CGGA RNA-seq database (*n* = 308) and Ditan Hospital database (*n* = 37) were divided into two groups based on the median expression of HOXB7. Figures 3A,B shows that in both all grade and LGG, patients in the low *HOXB7* expression group achieve a significantly longer survival time than their high expression counterparts (*p* < 0.0001, *p* = 0.0047, respectively). However, this association was not reproduced in conventional statistical significance in Grade IV (GBM) group (*p* = 0.0535, Figure 3C). Furthermore, the above results were also confirmed in the patients from the Ditan Hospital database (Figures 3D–F).

Next, we focused on the lower grade glioma patients. Survival curves showed that HOXB7 high expression subgroup, *IDH1* wildtype subgroup and 1p/19q non-codeletion subgroup had similar survival trends (*p* > 0.05, Supplemental Digital Content, Supplementary Figures 1A,B). The same result was also confirmed in the patients from the Ditan Hospital database (*p* > 0.05, Supplemental Digital Content, Supplementary Figures 1C,D).

### Homeobox B7 Expression Revealed as a Prognostic Factor in All Grade and Lower Grade Gliomas

**FIGURE 4:**
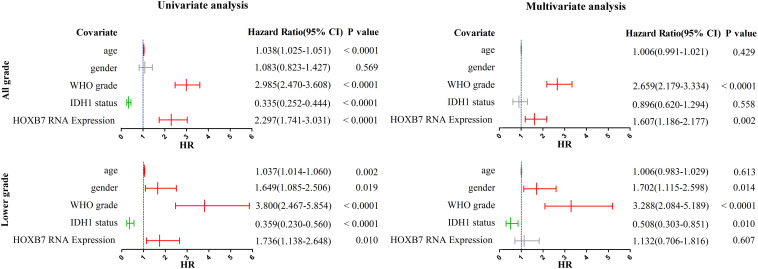
Univariate and multivariate analyses for overall survival (OS) in CGGA RNA-seq cohort (CI, confidence interval).

We subsequently performed univariate and multivariate analyses, including both clinicopathological and molecular features. In the all-grade group, univariable analysis based on the CGGA RNA-seq database identified age, WHO grade, IDH1 status and *HOXB7* expression as significant prognostic factors for specific survival (*p* < 0.0001, Figure 4). In the multivariable analysis, WHO grade and *HOXB7* expression were identified as independent prognostic factors (*p* < 0.0001, *p* = 0.002, respectively). In the lower grade group, the results of the univariable analysis showed that all five covariates were significant prognostic factors for OS (*p* = 0.002, *p* = 0.019, *p* < 0.0001, *p* < 0.0001, and *p* = 0.010, respectively). In the multivariable analysis, gender, WHO grade and IDH1 status were identified as independent prognostic factors (*p* = 0.014, *p* < 0.0001, and *p* = 0.010, respectively), although the significance of *HOXB7* expression was marginal (*p* = 0.607).

**FIGURE 5:**
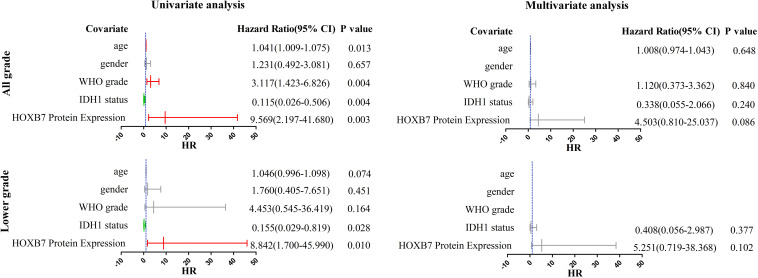
Univariate and multivariate analyses for OS in Ditan Hospital cohort (CI, confidence interval).

The results observed above were broadly consistent with the data from the Ditan Hospital database (Figure 5). In both the all grade group and the lower grade group, the results of the univariable analysis showed that HOXB7 protein expression was a significant prognostic factor for OS (*p* = 0.003 and 0.010, respectively). However, the significance of HOXB7 protein expression was only marginal (*p* = 0.086 and 0.102, respectively) in the multivariable analysis due to the small number of samples.

### Homeobox B7 Protein Deletion Is a Significant Feature of Oligodendroglioma

**FIGURE 6:**
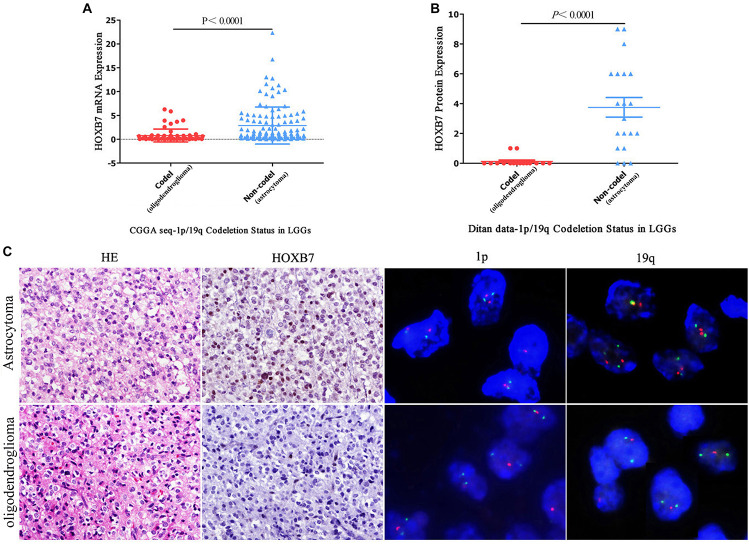
Homeobox B7 expression is related to 1p/19q molecular subtypes in CGGA and in Ditan data sets. The expression of HOXB7 is significantly lower in 1p/19q codeletion gliomas (oligodendroglioma) based on the CGGA data sets **(A)** and Ditan data sets **(B)**. Immunohistochemical staining of HOXB7 protein expression according to 1p/19q status showing the loss of HOXB7 protein expression in 1p/19q codeletion anaplastic oligodendroglioma. Only a few nuclei are weakly positive in the background. The other case of anaplastic astrocytoma, 1p/19q non-codeletion, showing the strong nuclear location of HOXB7 protein [**(C)**, HE, EnVision, serial sections, Original magnification ×400, FISH, Original magnification ×1,000].

As shown in Table 1, it is interesting to find that HOXB7 protein was negative in 20 of 22 cases of oligodendrogliomas (including WHO grade II and grade III) but in 23 cases of astrocytomas (including WHO grade II and grade III), there was a loss of HOXB7 protein expression in only 3 cases. Taking these two types of gliomas alone, the sensitivity and specificity of HOXB7 protein deletion in oligodendroglioma were 90.9% (20/22) and 87.0% (20/23), respectively. According to the 2016 WHO guidelines, 1p/19q is a molecular diagnostic marker for these two subtypes of gliomas. Therefore, we analyzed the correlation between HOXB7 RNA level and 1p/19q deletion status based on the RNA SEQ information of 325 cases of gliomas in the CGGA data sets. The results showed that the expression of HOXB7 mRNA was significantly lower in 1p/19q codeletion gliomas (oligodendroglioma) (*p* < 0.0001, Figure 6A). The trends of protein and mRNA expression levels are consistent, based on the Ditan data sets (*p* < 0.0001, Figure 6B).

The 2016 WHO guidelines recommend that oligodendrogliomas should be diagnosed by morphology, *IDH1* mutant and 1p/19q codeletion status. In most cases it is not easy to differentiate by morphology and IHC alone. As shown in Figure 6C, both histological images exhibit perinuclear halo like tumor cells, which if taken in isolation, render it difficult to make a definitive diagnosis of oligodendroglioma. When combined with 1p/19q codeletion detection, a diagnosis can be made with a greater degree of confidence. HOXB7 IHC staining may also be helpful. Although two oligodendrogliomas were positive for HOXB7 protein, the number of positive tumor cells and the intensity of positive signal were limited. The SQS values of IHC staining were 2 and 1, respectively, (Supplemental Digital Content, Supplementary Figure 2), which indicate weakly positive results. The other 20 cases were different, in that the staining was completely negative. Based on this limited information, we can infer that the diagnosis of oligodendroglioma is more appropriate if the HOXB7 IHC staining of one diffuse glioma was entirely negative.

Finally, to verify the reliability of HOXB7 in differentiating oligodendrogliomas from astrocytomas, we used 1p/19q FISH testing as a positive control. The consistency of IHC and FISH in the diagnosis of oligodendroglioma was further analyzed. The Cohen’s Kappa value was 0.778 (95% CI, 0.594–0.962), which implied that the two methods have a high level of concordance (*p* < 0.0001, Table 2). Taken together, these results suggest the utility of HOXB7 as a novel marker to differentiate oligodendroglioma from astrocytoma.

**TABLE 2:** Consistency test between 1p/19q Codeletion and HOXB7 protein loss in LGG.

Parameters		Total	IHC HOXB7 protein loss		FISH 1p/19q codeletion		Kappa value	*P*-value
		*n* = 45	YES (*n*)	NO (*n*)	YES (*n*)	NO (*n*)		
Oligodendrocytoma		22	20	2	22	0	0.778	<0.0001
WHO II	IDH mut	9	9	0	9	0		
	IDH wt	0	0	0	0	0		
WHO III	IDH mut	13	11	2	13	0		
	IDH wt	0	0	0	0	0		
Astrocytoma		23	2	23	0	23		
WHO II	IDH mut	8	2	6	0	8		
	IDH wt	2	0	2	0	2		
WHO III	IDH mut	5	0	5	0	5		
	IDH wt	8	1	7	0	8		

*n, number of cases; wt, wild type; mut, mutant; LGG, lower grade glioma.*

## Discussion

Homeobox B7 is highly expressed in embryonic tissues. During early embryonic development, HOXB7 is widely expressed in mesoderm, hindgut and central nervous system, while in late pregnancy, it is limited to specific tissues of the embryo. In normal adult tissues, HOXB7 is only expressed in thymus, tonsil, cerebellum, liver, endometrium, and cervix (Liedtke et al., 2010). *In vitro* and *in vivo* studies have demonstrated that HOXB7 dysregulation may play an important role in a variety of diseases including cancer. It is aberrantly expressed in a variety of cancers, including melanoma (Carè et al., 2001), colorectal cancer (Kanai et al., 2010), hepatocellular carcinoma (Huan et al., 2017), breast cancer (Heideman et al., 2015), esophageal squamous cell carcinoma (Zhou et al., 2020), intrahepatic cholangiocarcinoma (Dai et al., 2019), gastric cancer (He et al., 2017), pancreatic cancer (Chile et al., 2013), and other tumors. However, there are few reports on the relationship between HOXB7 and gliomas. Molecular genetic research (Sehgal, 1998) suggests that the occurrence of glioma is closely related to chromosome 9p, 10q, 17p, and 22q; the HOXB family happens to be located on chromosome 17.

In this study, HOXB7 was highly expressed in GBM and *IDH1* wild type gliomas at both mRNA and protein levels. *IDH1* wild type GBM indicates higher malignancy and shorter survival time in glioma (Louis et al., 2016). High HOXB7 expression is significantly positively correlated with GBM and *IDH1* wild type status, which indicates that HOXB7 functions as an oncogene in gliomas. Moreover, HOXB7 mRNA and protein levels are significantly down regulated in 1p/19q codeletion gliomas. The patients with HOXB7 high expression, *IDH1* wild-type and 1p/19q non-codeletion subgroups all had similar survival trends, which further illustrate the association between HOXB7 and *IDH1* wild-type gliomas, as well as 1p/19q non-codeletion gliomas.

Involvement of HOXB7 in known pathways is critical to the initiation and progression of other solid cancers including epithelial cells toward a more mesenchymal phenotype (EMT), angiogenesis, apoptosis, proliferation, migration, DNA repair and so on (Errico et al., 2016). In addition, a number of epigenetic regulatory mechanisms have been reported to be associated with HOXB7, such as changes in methylation status and histone posttranslational modifications, microRNA-based targeting and long non-coding-RNAs (lncRNAs) modulation (Zhang et al., 2014). It was surprising to find that HOXB7 protein was completely negative in almost all oligodendrogliomas in our study. We do not know the specific mechanism of HOXB7 involved in the occurrence and development of oligodendroglioma. There may be a point mutation or deletion of HOXB7, which results in the decrease of mRNA level in oligodendroglioma; alternatively the loss of protein expression could be caused by post-translational modification. Further studies are needed to evaluate this hypothesis.

Nearly all lower-grade gliomas with *IDH* mutations and no 1p/19q codeletion had mutations in *TP53* (94%) and *ATRX* inactivation (86%) (Cancer Genome Atlas Research Network, Brat et al., 2015). In contrast, *TP53* mutations are less common in gliomas with oligodendroglial features (9–44%) (Tanboon et al., 2016). Because *ATRX* mutations are uncommon in *IDH*-mutant tumors with 1p/19q codeletion, it is suggested that *ATRX*, along with *TP53* mutations, can be used as markers of astrocytic lineage. Concerned that some institutions would not be able to conduct a comprehensive molecular testing, cIMPACT Working Committee recommended (Louis et al., 2018) that 1p/19q testing can be omitted if an IDH-mutant tumor appears clearly astrocytic and the ATRX/p53 immunohistochemistry results are consistent with an astrocytic genotype (ATRX and/or TP53 mutations). Unfortunately, there are no standard criteria for what constitutes loss of ATRX staining in gliomas. Some studies imply *ATRX* mutations based on a single strict criterion; that is, no staining of any tumor nuclei. Others use less-strict cutoffs, for example, <10, <15%, or even <50% of tumor nuclei that stain for ATRX (Tanboon et al., 2016).

In this study, we found that HOXB7 may have utility as a novel marker to differentiate oligodendroglioma from astrocytoma. To the best of our knowledge, this is the first time this has been reported in the literature for diffuse gliomas. HOXB7 is more sensitive than ATRX and more specific than TP53. Furthermore, HOXB7 IHC staining and 1p/19q FISH detection have a high level of concordance in the diagnosis of oligodendroglioma. Therefore, staining for HOXB7 is recommended as the first-line marker along with TP53 and ATRX for molecular evaluation of diffuse gliomas, owing to advantages of immunohistochemistry over complex genetic studies.

The 2016 WHO guidelines (Louis et al., 2016) state that the prognosis of *IDH* wild-type diffuse astrocytoma is worse than that of the *IDH* mutant. The overall prognosis of some WHO II or III *IDH1* wild-type astrocytoma patients is poor, and the survival time is equivalent to or slightly longer than that for *IDH* wild-type glioblastoma patients. Therefore, *IDH1* mutation status alone is not enough to predict the prognosis of astrocytoma. With the development of sequencing technology, various studies have focused on the molecular stratification of gliomas to identify the types with different prognoses. CHI3L1 and NTRK2 were identified as factors that can be associated with *IDH1* status and 1p/19q codeletion to distinguish between prognostic groups of glioma from the TCGA cohort (Deluche et al., 2019). It appears to be a subgroup of *IDH1* mutated astrocytomas harboring 19q loss and *TP53* mutation, which can present morphology similar to oligodendroglial tumors, and also show significantly better prognosis compared to other astrocytomas with *IDH* mutation (Otani et al., 2018). Based on a large number of retrospective publications, the cIMPACT Working Committee concluded that amplification of epidermal growth factor receptor (*EGFR*) gene, and/or increase of whole chromosome 7 with loss of whole chromosome 10 (+7/−10) and/or mutation of TERT promoter are highly specific indicators for poor prognosis of *IDH* wild-type diffuse astrocytoma (Wijnenga et al., 2017).

In the current study, we utilized two independent cohorts to evaluate and validate the role of HOXB7 in prognosticating patients with glioma. Survival curve analysis showed that high HOXB7 expression confers a reduced survival time in all grade glioma and lower grade glioma patients. Although HOXB7 could not be used as a significant marker for further molecular stratification of LGG through our experiments. We noted that in lower grade glioma patients, the survival time with high expression of HOXB7 subgroup was similar to that of the *IDH1* wild type subgroup. Further multivariate Cox regression analyses showed that HOXB7 was an independent predictive factor of poor prognosis in all grade glioma patients.

In conclusion, HOXB7 is a robust diagnostic marker at differentiating between oligodendroglioma and astrocytoma with good sensitivity and specificity. Diffuse glioma patients, especially LGG with high-HOXB7 expression have a significantly worse prognosis, which is similar to that of the *IDH1* wild type, indicating that HOXB7 could play an important role in the prognosis estimation of diffuse glioma. Further large multicenter studies are needed to confirm our findings and determine its usefulness in the clinical setting.

## Data Availability Statement

The datasets presented in this study can be found in online repositories. The names of the repository/repositories and accession number(s) can be found in the article/Supplementary Material.

## Author Contributions

XZ and TL conceived and designed the experiments. JD and KN performed the language editing. ML and CL collected the case materials. JC, KY, and ZM performed the experiments. WM analyzed the data. PW wrote the manuscript. All authors gave final approval of the manuscript.

## Conflict of Interest

The authors declare that the research was conducted in the absence of any commercial or financial relationships that could be construed as a potential conflict of interest.

## Publisher’s Note

All claims expressed in this article are solely those of the authors and do not necessarily represent those of their affiliated organizations, or those of the publisher, the editors and the reviewers. Any product that may be evaluated in this article, or claim that may be made by its manufacturer, is not guaranteed or endorsed by the publisher.

## Footnotes

1
http://www.cgga.org.cn

## Supplementary Material

Supplementary Figure 1 The survival trends of HOXB7 high expression group in lower grade gliomas. **(A,B)** CGGA RNA-seq database; **(C,D)** Ditan database.

Supplementary Figure 2 The expression of HOXB7 protein is weakly positive in the hot field of two cases of oligodendrogliomas. The medium positive tumor cells are located in the lower right corner of case 1. The semi-quantitative scoring of HOXB7 protein expression is 2. The small number of weakly positive tumor cells scattered in case 2. The semi-quantitative scoring is 1 (HE, EnVision, serial sections, Original magnification ×400).
