# Supplementary figures and images for: Differential and Prognostic Significance of HOXB7 in Gliomas
Source: Front Cell Dev Biol. 2021 Aug 11;9:697086. doi: 10.3389/fcell.2021.697086 (PMC8385304; doi:10.3389/fcell.2021.697086)

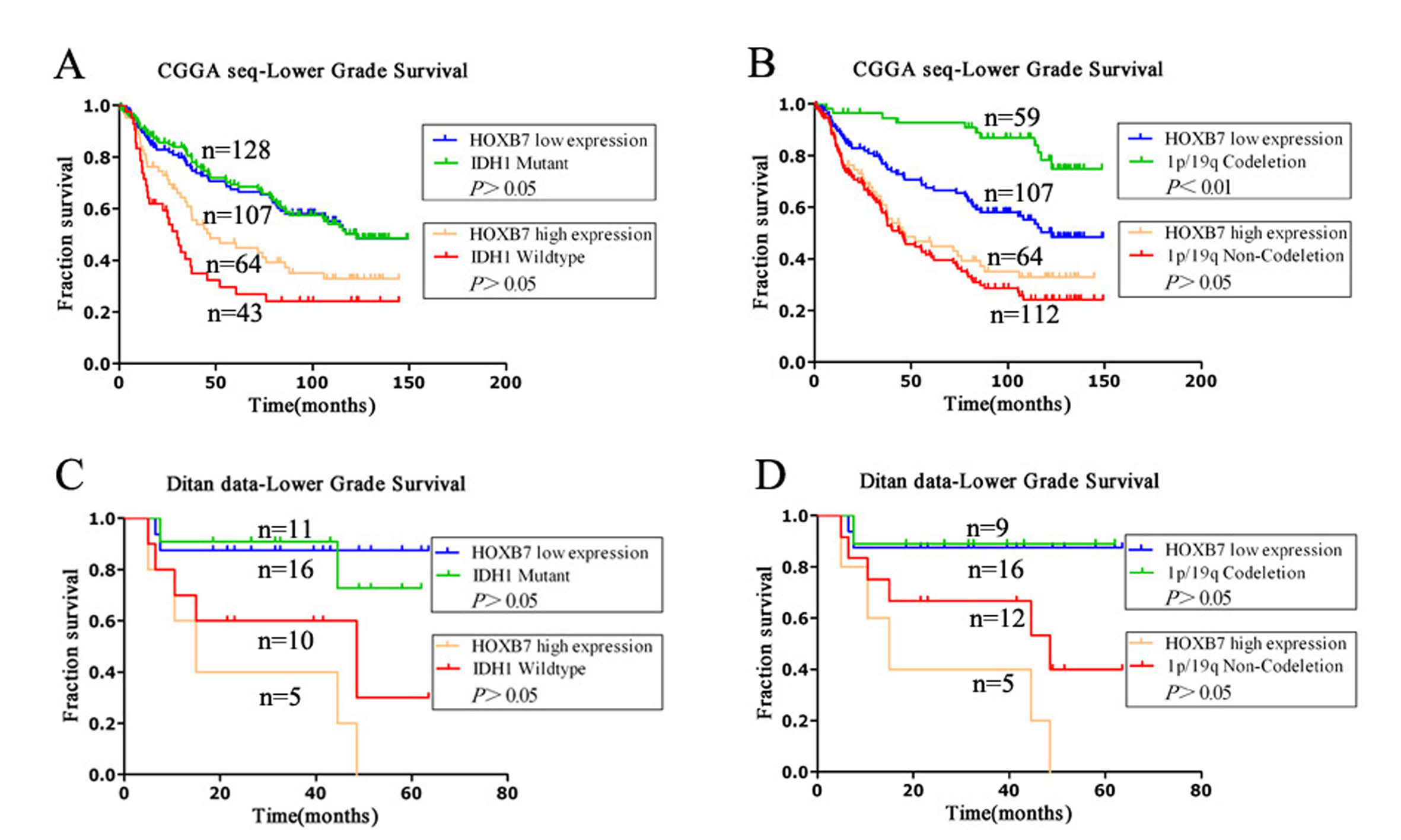

Supplement: Supplementary Figure 1 — The survival trends of HOXB7 high expression group in lower grade gliomas. (A,B) CGGA RNA-seq database; (C,D) Ditan database. [file Image_1.JPEG]

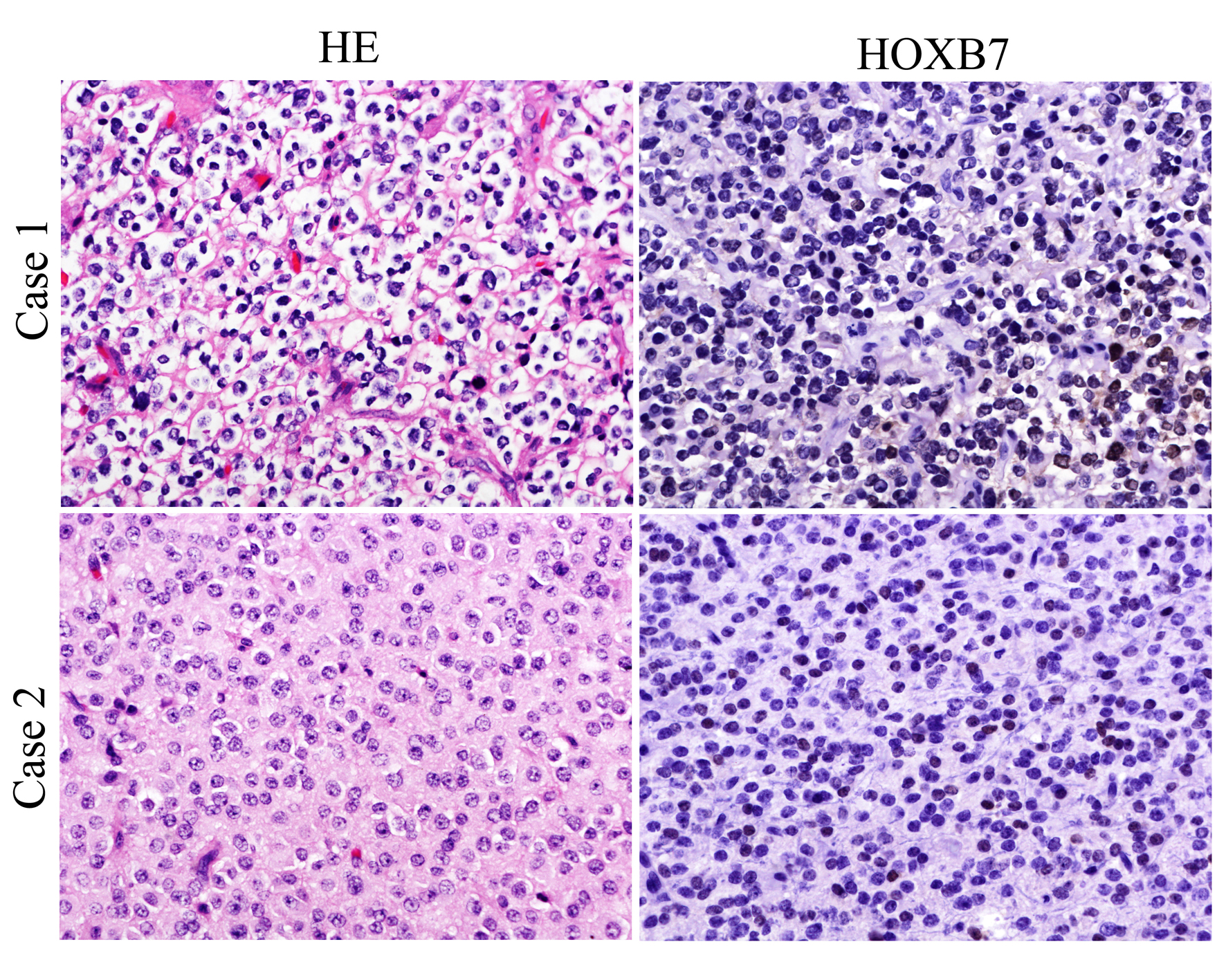

Supplement: Supplementary Figure 2 — The expression of HOXB7 protein is weakly positive in the hot field of two cases of oligodendrogliomas. The medium positive tumor cells are located in the lower right corner of case 1. The semi-quantitative scoring of HOXB7 protein expression is 2. The small number of weakly positive tumor cells scattered in case 2. The semi-quantitative scoring is 1 (HE, EnVision, serial sections, Original magnification ×400). [file Image_2.JPEG]
